# How Can We Witness the Publication of Ethical Research in the Future? A Viewpoint

**DOI:** 10.34172/ijhpm.9254

**Published:** 2025-09-20

**Authors:** Sara Dakhesh, Shahnaz Khademizadeh, Abdolhossein Farajpahlou, Hamid Farhadirad

**Affiliations:** ^1^Department of Medical Library and Information Sciences, School of Allied Medical Sciences, Tehran University of Medical Sciences, Tehran, Iran.; ^2^Department of Knowledge and Information Science, Shahid Chamran University of Ahvaz, Ahvaz, Iran.; ^3^Department of Educational Science, Faculty of Education and Psychology, Shahid Chamran University of Ahvaz, Ahvaz, Iran.

## Introduction

 The growth of scientific production has led to an increase in the rate of article retractions, highlighting the need for special attention to the key principles that uphold and advance the integrity of science. Scientific misconduct diverts the progress of research away from a healthy and dynamic path, undermining the credibility of scientific institutions and creating barriers to societal development.^1^

 The main issue is that, despite various actions and policies implemented by research systems, we still face challenges related to insufficient adherence or, in some cases, non-compliance with the ethical standards of publication in scientific works.^2^ Research systems have predominantly focused on controlling or detecting scientific misconduct, but these measures have proven to be insufficiently effective. Given the critical nature of the scientific publication cycle, it is essential to complement these oversight efforts by identifying both internal and external factors that influence future trends. Understanding these factors will help recognize potential opportunities and threats, allowing for better prevention and management of issues at both the organizational and individual levels. This proactive approach can lead to scientific excellence and foster a positive future in publication ethics. Achieving publication ethics requires more than a reactive stance; by implementing optimal strategies, the scientific community can be guided toward adherence to ethical standards in publication.^3^

 Given the importance of research systems and the essential roles of universities, research organizations, information centers, and libraries, it is crucial to develop solutions for establishing the necessary infrastructure. Additionally, publishers of scientific works should be encouraged to adhere to research policies that align with publishing ethics. Adhering to these standards by all participants in the research system—including researchers, editors, publishers, and journal reviewers—as well as policy-makers and decision-makers, will be invaluable. This adherence will promote an increase in citations of published articles and attract both human and economic resources to research, ultimately enhancing the scientific indicators of countries.

 In light of these considerations, it is crucial to lay the foundation for developing dedicated and skilled human resources and to create a desirable future that aligns with the principles of publishing ethics. Foresight in the field of publishing ethics, while activating warning systems, engaging various stakeholders and experts in decision-making, identifying key technologies, removing current limitations, and educating and informing policy-makers, will lead to improved decision-making and stronger policy-making results.^4^ In this regard, the Global Foresight Group has stated that the days of relying solely on statistical predictions are over; instead, we must adopt a more comprehensive perspective and use foresight techniques to broaden the scope of information processing.^5^ The increase in scientific misconduct in publishing research results shows that the regulatory and control approaches provided by laws and ethical guidelines in publishing have been inadequate. Additionally, various studies so far have only identified the factors that influence the overall improvement of research ethics. It is essential to concentrate on the most critical stage of the research process: The timely and accurate publication of results. We need to develop and present strategies in the form of desirable future scenarios that address existing challenges in publishing ethics. These strategies should adopt a long-term, participatory, and problem-solving approach. Current issues serve as a cautionary signal for planning and considering the future. It is crucial to consider publication ethics in research policy-making to promote the dissemination of ethical research.

## Key Factors Shaping the Future of Publication Ethics in Research

 In today’s world, science, technology, and ethics are more interconnected than ever. This indicates that while new advancements have led to the adoption of modern technologies in various research methodologies, science and research must adhere to established standards and values; they cannot operate independently from ethics. Furthermore, with the rise of scientific misconduct and the increasing number of article retractions, it is crucial to identify the factors influencing publication ethics. Research entities need to implement necessary measures to manage and control these factors within the future research system. The prevalence of research misconduct undermines scientific authority and can have irreversible effects on the scientific credibility of nations.

 By examining publication ethics in the policy-making of the research system, it was found that several factors across seven key areas—political, economic, social, technological, institutional/organizational, ethical, and legal—impact the future of publication ethics in research policy-making. These factors are categorized as influential driving forces,^6^ as illustrated in Figure.

**Figure:**
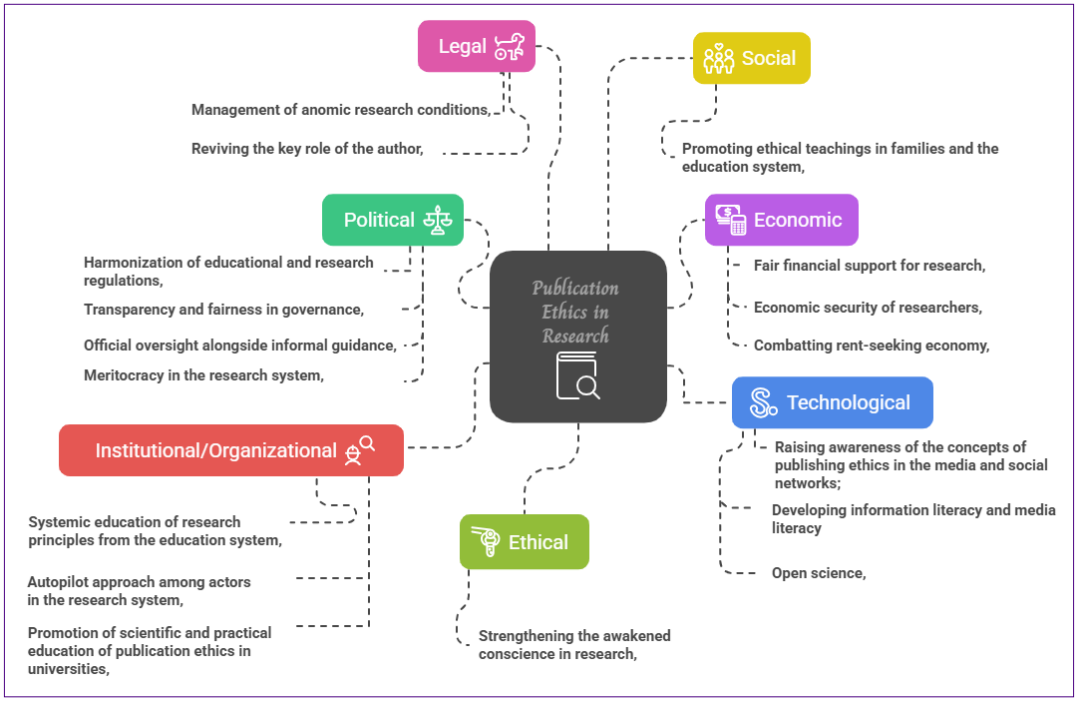
Influential Forces on the Future of Publication Ethics in Research.

## The Ideal Scenario for the Future of Publication Ethics in the Research System

 The ideal scenario for publication ethics envisions a future where policy-makers clearly define the needs and research priorities of countries, integrating the principles of science and research. In this future, researchers will have a better understanding of the underlying philosophy of research and will be more inclined to conduct studies that are focused on solving real-world problems and addressing specific demands. Implementing such measures will enhance the perspectives and actions of research participants, helping to reduce both unintentional and intentional violations during the publication of results. Additionally, these participants gain valuable ethical guidance from educational institutions, including families and schools, which fosters a strong research culture. As a result, researchers develop a deep understanding of ethical principles and a heightened awareness that compels them to adhere to these standards. With a vigilant conscience that resists environmental pressures, they are less likely to engage in research misconduct.

 The primary goal of this ideal scenario in policy-making is to establish and promote meritocracy within the research system. By valuing fundamental, applied, and problem-oriented research, this ethical research framework fosters an environment that encourages and supports individuals committed to ethical principles. As a result, researchers and other stakeholders in the research community will feel motivated to publish their scientific work in adherence to publication ethics. In another aspect of this scenario, efforts will be made to build an ideal future based on publication ethics. This will involve using empowering technologies, media, and social networks to promote ethical standards and codes, highlight instances of scientific misconduct, and provide information about the laws and regulations governing research and publication ethics. These initiatives can enhance the ethical literacy of researchers and support formal oversight in achieving ethical objectives in research. Notably, the implementation and updating of intelligent monitoring software will be effective in preventing and addressing numerous research violations.

 Therefore, although there are many uncertainties regarding the future, it is crucial to establish an ethical research system by making timely decisions and developing effective strategies. Policy-makers at the macro level play a vital role in this process. One important action is to grant sufficient autonomy to universities and higher education institutions. Currently, many research and educational regulations and guidelines are shaped by the political and economic objectives of various countries. This situation inadvertently places pressure on researchers and impacts the quality of their outputs. To address these challenges, research and academic activities need to occur in an independent environment, free from political influences and economic pressures. In this context, intermediary research entities—such as information centers, academic communities, associations, and libraries—play a crucial role in promoting self-sustaining models and fostering independent thinking among researchers.

 If universities have sufficient independence, we are likely to see the emergence of networks of leading scholars and scientific experts within academic associations. These networks will focus on raising awareness, sharing knowledge and experiences, and teaching ethical concepts and values to the scientific community. As a result, a healthy research culture will become deeply ingrained in the attitudes and behaviors of researchers, leading to a reduction in the reliance on punitive laws and legal regulations to control and oversee research activities.

 Research and educational regulations will be most effective when they not only clarify the legal aspects of research but also introduce the fundamental pillars, principles, and standards of research ethics and publication. By doing so, we can foster ethical literacy within the scientific community and, in turn, increase researchers’ commitment to the principles of research and publication ethics.

## Strategies for Attaining an Ideal Future of Publication Ethics in the Research System

 To establish ideal publication ethics, experts in this field have suggested various strategies. In reviewing these recommendations and consolidating key points, we will present the relevant items here:

1. Strengthening evaluation and validation systems for research by avoiding a focus on evaluating and ranking the research performance of universities and research centers solely based on quantitative indicators, and instead concentrating on the impact of research across various social, economic, political, health, environmental, and other dimensions.
2. Valuing true excellence in various fields of the research system through the provision of grants, study opportunities, and employing them in important, impactful, and relevant executive positions related to research areas.
3. Conducting continuous training courses on research ethics and publication ethics.
4. Formulating supportive and incentive policies regarding adherence to publication ethics.
5. Setting up deterrent social, organizational, and legal regulations regarding violations of publication ethics, aligned with the laws of the International Committee on Publication Ethics.
6. The necessity of completing courses on research ethics and publication as a mandatory prerequisite for starting the thesis process.
7. The necessity of participating in training courses on research ethics and publication as a mandatory prerequisite for implementing national and international research projects.
8. Establishing specialized intellectual property courts in various centers.
9. Publishing the guidelines of the International Committee on Publication Ethics on the websites of journals and the research and technology departments of universities and research centers.
10. Adjusting the weight of points awarded for publishing scientific works and weighting other scientific, educational, and research capabilities in the academic system.
11. Highlighting the role of ethics committees in universities and research centers, and obtaining commitments from researchers in the form of an ethical commitment letter before conducting and publishing research.
12. Establishing a coherent system for reviewing scientific works (articles, theses, and books) in educational and research centers.
13. Reviewing and renewing educational and research regulations.
14. Developing internal coherence in the components of evaluating educational and research guidelines.
15. Establishing and incorporating courses on the ethics of science, writing ethics, information ethics, research ethics, and publication ethics into the educational system.
16. Revoking all academic privileges and benefits for individuals whose scientific misconduct in their works has been proven.
17. Creating appropriate mechanisms for the continuous monitoring of research activities aimed at reducing unintentional/intended misconduct.
18. Developing a suitable process for monitoring reporting systems for research misconduct and retraction of articles.
19. Extensive support for problem-oriented and demand-driven research within the research system.
20. Support for whistleblowers and individuals who identify and report corruption bottlenecks in research fields, and the establishment of a system for receiving reports from whistleblowers regarding scientific misconduct.
21. Networking knowledge related to publication ethics in such a way that each of the leaders in this field takes on the responsibility of guiding and leading the members.
22. Institutionalizing a culture of self-regulation, inquiry, critical and creative thinking, teamwork, and a research-oriented culture instead of a memory-oriented culture among the actors in the higher education system.
23. Overcoming financial challenges and increasing the share of research budgets from gross domestic product.
24. Research mission orientation and delegating specific research missions on particular topics to universities.
25. Encouraging mass media such as state television and other public networks and media to include effective content and programs regarding the awareness of publication ethics concepts, aiming to strengthen the awakened conscience in society.
26. Presenting an ideal scenario for the future of publication ethics to policy-makers in the educational and research systems.

## Practical Suggestions

 Ultimately, suggestions have been made to achieve an ideal scenario for the future publication ethics system of societies:

1. Policy-makers in the educational sector, particularly vice presidents and educational managers, are focusing on strengthening specialization within universities and higher education institutions. To achieve this, they should engage leading figures and esteemed scientists to help foster a research culture within academic communities and associations. By delegating authority to universities and higher education institutions, we can promote greater independence in the education and research systems, ultimately enhancing the quality of various fields of study at the national level. Another crucial step is to develop scientific authority in the realm of ethical sciences through the educational system. The objective is to introduce key individuals to the scientific community at both national and international levels, thereby creating a network of specialists in ethical sciences. This network will help cultivate an environment that promotes a healthy and respectable research culture among students and professors alike.
2. In the main section, which includes policy-makers, planners, and decision-makers in the research system—specifically within the Office of Research Planning and Policy and the Office of Support and Assistance for Research Affairs—there is a pressing need to review and update regulations and guidelines. This would help create coherence and consistency in the formulation of educational and research policies. Additionally, it is essential to develop supportive policies that honor and celebrate ethical scholars within the research system. This effort should also include plans for renewing and transforming the ethical standards of young scientists, thereby enhancing their motivation for ethical conduct. Another important aspect for the Research Department is the necessity for comprehensive, extensive, and continuous oversight of scientific journals. To achieve this, conducting annual and regular assessments to evaluate the awareness and performance of editors, editorial board members, reviewers, and publishers concerning ethical codes in research would be highly beneficial.
3. Policy-makers in the Technology and Innovation Department should collaborate with skilled technology teams from knowledge-based companies and accelerators within research and growth centers. Together, they can design and implement advanced monitoring and management software to ensure the quality of scientific publications in journals.

It is crucial to emphasize that all proposed suggestions necessitate enhancing the foresight knowledge of high-level stakeholders, such as policy-makers, managers, planners, and decision-makers. By taking these actions, it will be possible to achieve the desired scenario of promoting ethics in the future of research systems through press conferences and policy initiatives.

## Acknowledgements

 We express our gratitude to the valuable insights of the esteemed panel members, interviewees, and subject matter experts who contributed to the completion of this work.

## Ethical issues

 Not applicable.

## Conflicts of interest

 Authors declare that they have no conflicts of interest.
